# Predicting the Impact of Climate Change on the Distribution of a Neglected Arboviruses Vector (*Armigeres subalbatus*) in China

**DOI:** 10.3390/tropicalmed7120431

**Published:** 2022-12-12

**Authors:** Gang Wang, Dongjing Zhang, Jehangir Khan, Jiatian Guo, Qingdeng Feng, Yan Sun, Beiqing Li, Yu Wu, Zhongdao Wu, Xiaoying Zheng

**Affiliations:** 1 Department of Parasitology, Key Laboratory of Tropical Disease Control of the Ministry of Education, Sun Yat-sen University, Guangzhou 510080, China; 2 CAEA Center of Excellence on Nuclear Technology Applications for Insect Control, Sun Yat-sen University, Guangzhou 510080, China; 3 SYSU Nuclear and Insect Biotechnology Co., Ltd., Dongguan 523000, China; 4 Guangdong Provincial Engineering Technology Research Center for Diseases-Vectors Control, Sun Yat-sen University, Guangzhou 510080, China; 5 IAEA Collaborating Centre for Developing the Sterile Insect Technique (SIT) for Control of Mosquito, Sun Yan-sen University, Guangzhou 510080, China; 6 Department of Zoology, Abdul Wali Khan University Mardan, Mardan 25000, Pakistan

**Keywords:** Maxent, future potential distribution, *Armigeres subalbatus*, arboviruses vector, climate change

## Abstract

The geographic boundaries of arboviruses continue to expand, posing a major health threat to millions of people around the world. This expansion is related to the availability of effective vectors and suitable habitats. *Armigeres subalbatus* (Coquillett, 1898), a common and neglected species, is of increasing interest given its potential vector capacity for Zika virus. However, potential distribution patterns and the underlying driving factors of *Ar. subalbatus* remain unknown. In the current study, detailed maps of their potential distributions were developed under both the current as well as future climate change scenarios (SSP126 and SSP585) based on CMIP6 data, employing the MaxEnt model. The results showed that the distribution of the *Ar*. *subalbatus* was mainly affected by temperature. Mean diurnal range was the strongest predictor in shaping the distribution of *Ar*. *subalbatus,* with an 85.2% contribution rate. By the 2050s and 2070s, *Ar*. *subalbatus* will have a broader potential distribution across China. There are two suitable expansion types under climate change in the 2050s and 2070s. The first type is continuous distribution expansion, and the second type is sporadic distribution expansion. Our comprehensive analysis of *Ar*. *subalbatus*’s suitable distribution areas shifts under climate change and provides useful and insightful information for developing management strategies for future arboviruses.

## 1. Introduction

Mosquitoes are a major public health concern, because a number of their species can play a pivotal role in the transmission of a variety of pathogens. The wide geographical distribution and dispersal dynamics of mosquitoes partly shapes the entomological risk of vector-borne disease transmission internationally [1,2]. Climate changes can significantly influence the distribution and dispersal of mosquitoes [3,4]. Due to climate change, many species of mosquito have successfully expanded and established populations in many parts of the world due to their strong ability to colonize different microhabitats [5]. In fact, climate change is increasing the introduction of mosquitoes to occupy the suitable areas where they were previously absent [6]. Therefore, the change in mosquito distribution dynamics under the influence of climate change will bring new challenges to controlling the transmission of mosquito-borne diseases.

Current knowledge suggests that climate change and mosquito-borne diseases have a well-studied relationship [7]. As the climate changes, more areas may become suitable habitats for vectors in the future [8], and mosquito vectors will introduce diseases to people living in non-endemic or low-transmission areas [9]. Therefore, mosquitoes and the arboviruses they transmit will increase dramatically [10,11,12,13]. This brings huge challenges to the monitoring and early warning of the emergence and spread of arboviruses.

Zika virus (ZIKV) is a pathogen primarily transmitted by the bite of an infected female mosquito from the *Aedes* genus that poses a serious threat to global health in tropical and subtropical regions [14,15]. Previous experimental infection and transmission studies have proved that *Ae*. *aegypti* and *Ae*. *albopictus* are potential vectors of ZIKV [16,17,18,19,20]. Some research has shown that ZIKV may be transmitted by more than one vector [15,21]. The public health community now recognizes the potential threat posed by these two widespread invasive species (*Ae. aegypti* and *Ae. albopictus*) [22]. However, continued focus on a few important mosquito species as a major threat ignores other potential vectors in the expansion of arboviruses’ (arthropod-borne viruses) transmission, which could increase the risk of vector-borne disease outbreaks.

*Armigeres subalbatus* (Coquillett, 1898) has been confirmed as a new potential transmission vector of ZIKV recently [18,23,24,25]. These studies confirm that some important potential vectors may have remained unexamined and long-neglected. *Ar. subalbatus* is a common mosquito species in China [26] that has the potential to transmit several pathogens (such as filariasis and Japanese encephalitis virus) [27,28]. However, there has been no systematic research on *Ar. subalbatus*, resulting in many vacancies in its important ecological characteristic data (such as distribution data) that can be used to evaluate public health security. A few studies have proven that most of the neglected vectors are spreading geographically, creating a significant threat of mosquito-borne virus transmission [4,22]. Therefore, before formulating specific prevention and control strategies, future distribution forecasts based on current mosquito distribution data to assess their transmission risk are becoming particularly important and urgent.

In China, *Ar. subalbatus* is found primarily in the south of the Yangtze River [26]. However, the specific distribution data has not been obtained and studied. Moreover, the potential range of *Ar. subalbatus* has not yet been investigated, and it is uncertain how climate variables may affect its distribution pattern. This knowledge is crucial for the prevention and management of this significant pest [29]. Recently, species distribution modeling (SDM) was introduced to describe the ecological needs of a given species using environmental factors associated with occurrence data [30,31,32,33,34]. Further, SDM has been intensively used as the best tool with which to assess, quantify, and visualize the potential impacts of climate change on mosquitoes’ geographic expansion [35]. Many previous studies have shown the importance of modeling the distribution of vectors for risk assessments of vector-borne diseases [36,37,38]. The results of these studies can be used to guide the implementation of controlling programs for vector-borne diseases.

Knowing the current distribution of *Ar. subalbatus* and its possible shift in response to climate changes in the future is essential for comprehensive public health planning. Hence, we generated prediction models of *Ar. Subalbatus’* potential distribution in China under both present-day and future climate conditions (the 2050s and 2070s) using species distribution modeling.

## 2. Materials and Methods

### 2.1. Species Occurrence Data

**Figure 1:**
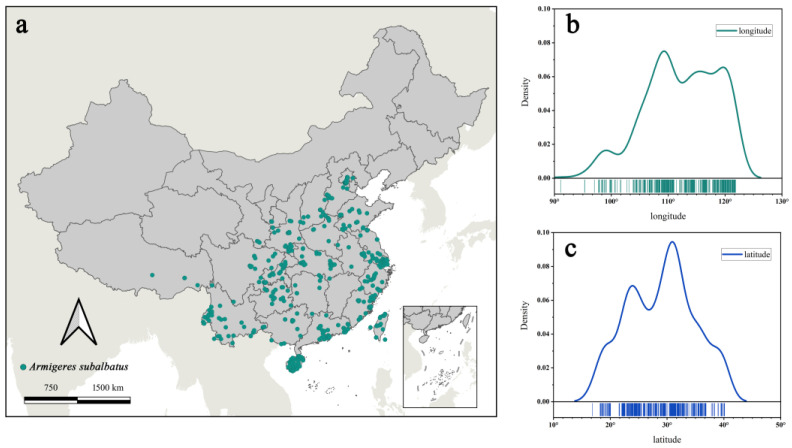
Overview of the observed occurrence records used for predictions. (**a**) The observed occurrence records of *Ar. subalbatus* in China; (**b**) longitudinal distribution curve of *Ar. subalbatus* habitat in China; (**c**) latitudinal distribution curve of *Ar. subalbatus* habitat in China.

*Ar. subalbatus* is a common mosquito species in the integrated regions between rural and urban areas of Asian countries [39]. The occurrence records of *Ar. subalbatus* were obtained from three frequently used sources: (1) published literature searched in Web of Science (https://www.webofscience.com/wos/alldb/basic-search, accessed on 1 August 2022) and CNKI (the Chinese National Knowledge Infrastructure, https://www.cnki.net/, accessed on 3 August 2022); (2) databases, including the GBIF (Global Biodiversity Information Facility, http://www.gbif.org, accessed on 3 August 2022) databases; and (3) field surveys during 2019 and 2021 in several provinces of China (including Shaanxi, Henan, and Guangdong). Initially, we received 531 presence records with exact coordinates (Figure 1).

The occurrence data obtained from web databases frequently contains sampling bias and has heterogeneous sampling intensities [40]. This will increase the over-representation of specific locations within a research area, resulting in a significant spatial bias in the occurrence data obtained [41,42]. Because those spatial relationships often lead to environmental bias, the difference between occurrence collecting and background sampling may result in erroneous models [43]. Thus, the function “Trim duplicate occurrences” of the ENMTOOLS software was then used to clean the occurrence records and reduce the potential spatial deviation caused by sampling bias [44]. The ENMTools tool can automatically match the size of the environmental factor grid used for analysis and delete redundant data in the same grid, thereby mitigating data sampling bias [44]. Afterwards, a data set with one record that occurred within each grid cell (5 × 5 km) can be obtained for follow-up analysis.

### 2.2. Environmental Predictor Variables

Two climate data sets were applied to summarize current and future climate conditions as environmental layers and cropped to the geographic area of China with a 5 × 5 km spatial resolution (2.5 arc-minutes). The data sets downloaded from PaleoClim (http://www.paleoclim.org/, accessed on 10 August 2022) for the Anthropocene v1.2b period (over the period 1979–2013) were regarded as the current environmental condition layer [45]. The other data sets downloaded from WorldClim version 2.1 (www.worldclim.org, accessed on 10 August 2022) were applied as the future periods’ environmental layers under different climate change scenarios [46]. Both datasets contain 19 bioclimatic variables that reflect monthly temperature and precipitation data obtained from climate stations across the world.

Predicting the future potential distribution of *Ar. subalbatus*, six different climate change scenarios were considered by combining the three global climate models (GCMs) (BCC-CSM2-MR, CNRM-CM6-1, and MIROC-6) with the two Shared Socio-Economic Pathways (SSPs) (SSP 126, green; SSP 585, high) [47]. Under each SSP, we used climate data from two periods (2041–2060, 2050s, and 2060–2080, 2070s) to project future habitat distribution changes. All operations were performed in QGIS v3.26.1 (https://www.qgis.org/en/site/, accessed on 23 July 2022).

Multicollinearity and dimensionality among bioclimatic variables will probably cause computational artefacts when analyzing species–environment relationships [48,49]. To reduce the impact of multicollinearity and dimensionality on the accuracy of the model fit, we process the bioclimatic variables as follows: (1) Due to known spatial distortions, the modelization excludes the bioclimatic variables Bio8 (mean temperature of the wettest quarter), Bio9 (mean temperature of the driest quarter), Bio18 (precipitation of the warmest quarter), and Bio19 (precipitation of the coldest quarter) [50,51]. (2) The multicollinearity and dimensionality between bioclimatic variables were reduced by performing the person correlation coefficient (PCC) and principal component analysis (PCA) with the remaining 15 environmental variables, respectively. Referring to the results of the PCA, only one bioclimatic variable was selected from a set of highly correlated variables (|r| ≥ 0.8), which was biologically important for *Ar. subalbatus* distribution [52]. The “corrplot” package and “factoextra” package in R were used to perform the PCC and PCA analysis. (3) The variance inflation factors (VIF) analysis was carried out to remove the highly correlated environmental variables, since their strong correlation reduced the accuracy of the model [38,53,54]. It was considered that variables with VIF values (greater than 10) had a multicollinearity issue [39]. The “USDM” package in R was used to analyze the VIF.

### 2.3. Species Distribution Modeling

The MaxEnt (maximum entropy algorithm), the most extensively used robust model for predicting species distribution, was used to predict the *Ar. subalbatus* potential habitat distribution under current and future climatic conditions. To improve the performance of Maxent and avoid overfitting, the ENMeval 0.2.1 R (version 4.1.3) package was employed to adjust the regularization multiplier (RM) and feature class (FC) parameters [55]. Model performance was evaluated based on the Akaike information criterion corrected (AICc) for small sample sizes.

The Maxent version 3.4.1 software [56] was used to develop the species distribution modeling (SDM) of *Ar. subalbatus* in different future climate scenarios. The jackknife method was utilized to determine the contribution of each bioclimatic variable. The distribution data were randomly divided into two parts: the test set (25% of the distributed data) and the training set (75% of the distributed data). The maximum number of iterations was set to 5000, and the maximum number of background points was set to 20,000. The model was repeated 10 times to obtain the average outputs.

The reliability of the models was evaluated according to four criteria, including values of AUC (area under the receiver operating characteristic curve) [56], the continuous boyce index (CBI) [57,58], AUC_DIFF_ [59], and the minimum training presence value (OR_MTP_) [60]. AUC values range from 0 to 1, with larger values indicating better model performance. The CBI has a range of −1 to 1, signifying inferior performance to a random model [61] and positive values indicating a good model performance. For models based on presence-only test data, this index is the most suitable assessment metric [61]. The CBI was implemented in the ecospat R package [62]. Based on the intuitive notion that overfit models should often perform well on training data but badly on test data, the metric (AUC_DIFF_) was developed. The larger the value of AUC_DIFF_, the more serious the overfitting of the model we built [59]. Additionally, based on the minimum training presence value (OR_MTP_), we estimated the test point omission rate to determine whether the best model is overfitting. This statistic ranged from 0 (not overfitted) to 1 (overfitted) and was threshold-dependent [60].

The probability (between 0 and 1) was applied to display the logical output of SDM, which can be explained as the suitability levels of *Ar. subalbatus* in the corresponding grid cell. The threshold of the MTSPS (Maximum Training Sensitivity Plus Specificity) was selected to define suitable and unsuitable regions of *Ar*. *subalbatus*. Based on this, the habitat suitability was divided into four degrees depending on the threshold value. The distribution probability less than the threshold value was considered unsuitable. These distribution probability values greater than the threshold and less than 1 are considered suitable, and they are equally divided into three levels (highly suitable areas, moderately suitable areas, and lowly suitable areas). The grid cells with suitability greater than 0.2368 were defined as potential suitable areas (wherein 0.7456–1 for highly suitable areas, 0.4912–0.7456 for moderately suitable areas, and 0.2368–0.4912 for lowly suitable areas).

### 2.4. Evaluation of Changes in Potential Suitable Areas

To effectively evaluate the impacts of various periods (2050s and 2070s) and SSPs (SSP126 and SSP585) on *Ar. subalbatus*, the means for the outputs of the four GCMs under the same SSP during the same time periods were computed. Under present and projected climatic scenarios, the distributions and geographic centroids of *Ar. subalbatus* were compared using the software SDMtoolbox [63].

## 3. Results

### 3.1. Observed Distributions and Climate Factors Selected

**Figure 2:**
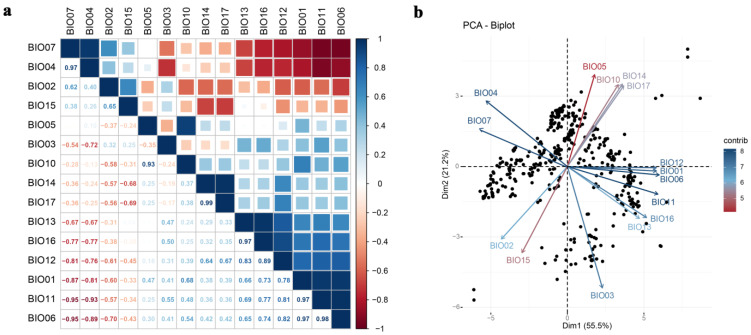
PCC (person correlation coefficient) and PCA (principal component analysis) of climatic factors. (**a**) Person correlation coefficient; (**b**) principal component analysis.

In total, observed occurrence data (531 presence records) were cleaned and yielded 431 unique occurrence records for *Ar*. *subalbatus*. Occurrence points for *Ar*. *subalbatus* were available from 26 provinces in China (Figure 1a). The northernmost area of *Ar*. *subalbatus* was close to the Yanshan Mountains. The longitudinal distribution of the *Ar*. *subalbatus* habitat ranged from 91°06′15″ N to 121°51′15″ N (Figure 1b). The latitudinal distribution of the *Ar*. *subalbatus* habitat ranged from 16°53′45″ E to 40°08′45″ E (Figure 1c). PCC results showed a high correlation between bioclimatic variables (Figure 2a). Bio12 was highly correlated with Bio6, Bio7, Bio9, Bio11, Bio13, and Bio16. Bio5 was also found to be highly correlated with Bio1, Bio8, and Bio10. The distribution of the 431 records was presented based on the first two main components (PCs), according to the PCA results (Figure 2b). The first two PCs accounted for 76.7% of the variance. The representative variables in PC1 were Bio2 and Bio11, and in PC2, they were were Bio5, Bio10, and Bio12 (Table 1). The results of the PCA and PCC were applied to the VIF as upstream data. Finally, it was decided to include Bio2, Bio3, Bio5, Bio12, Bio14, and Bio15 in the model based on the results of the VIF.

### 3.2. Model Performance

**Figure 3:**
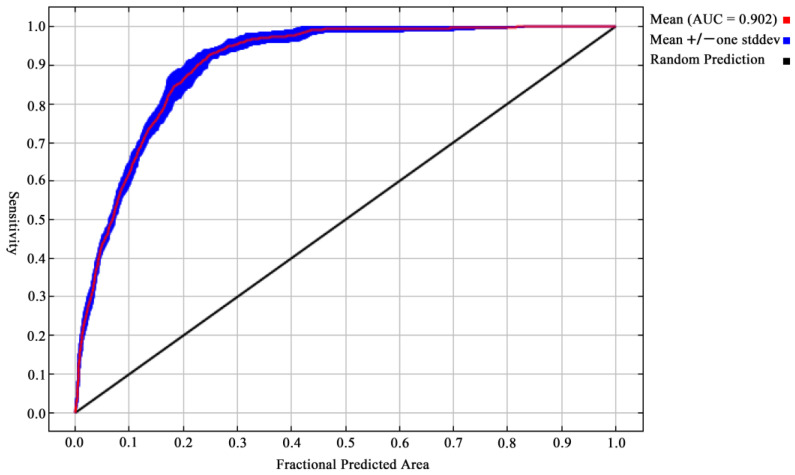
Receiver operating characteristic (ROC) curves and values of the area under the curves (AUC) of the modelling.

Employing the package ENMeval in R, the regularization multiplier and feature combination were calculated under the selected environmental variables [55]. The optimal FC for *Ar*. *subalbatus* was linear-quadratic (LQ) features, and the optimal RM was 1. Model performance was evaluated based on the Akaike information criterion corrected (AICc) for small sample sizes (ΔAICc = 0). Our model was acceptable as a sufficient representation of the *Ar*. *subalbatus* habitat’s suitability based on evaluation metrics (AUC = 0.9024 ± 0.0085 SD (Figure 3); mean CBI = 0.9100). Furthermore, the average OR_MTP_ value (0.0153) and the AUC_DIFF_ (0.0005) showed that our models were not overfitted.

### 3.3. Current Distribution of Suitable Habitat

**Figure 4:**
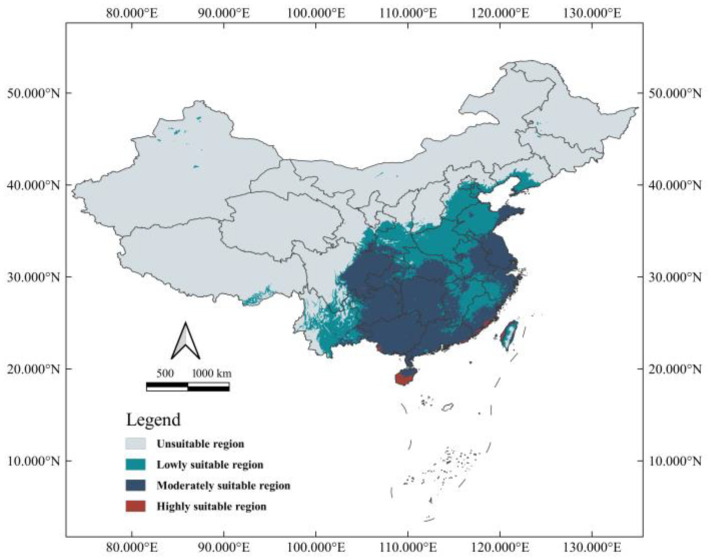
Modeled habitat suitability of *Ar*. *subalbatus* under current climate conditions in China.

Currently, suitable habitats for *Ar*. *subalbatus* are predicted to exist in all provinces except Qinghai in China (Figure 4). In addition to the most continuous suitable areas, there were also discontinuous suitable areas scattered in several provinces. Small parts of six provinces (Inner Mongolia, Liaoning, Jilin, Heilongjiang, Ningxia, and Tibet) were also considered suitable habitats for *Ar*. *subalbatus*. The whole area of suitable habitats is spread across 3,676,508 km^2^, which accounts for about 38.27% of the national territory of China.

### 3.4. Important Bioclimatic Variables

**Figure 5:**
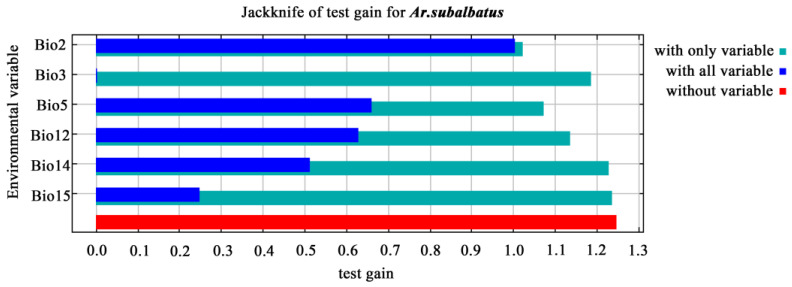
The key bioclimatic variables affecting the distribution of *Ar. subalbatus*. Bio 2: Mean diurnal temperature range (°C); maximum temperature of warmest month (°C) (Bio5, 9.7%); Bio15: precipitation seasonality; isothermality (°C) (Bio3, 1.2%), annual precipitation (mm) (Bio12, 3.5%), and precipitation of driest month (mm) (Bio14, 0.2%).

The mean diurnal range (°C) (Bio2, 85.2%) and maximum temperature of the warmest month (°C) (Bio5, 9.7%) contributed significantly to the model compared to other bioclimatic variables (Figure 5). Precipitation seasonality (Bio15, 0.1%) was the least important factor. Isothermality (°C) (Bio3, 1.2%), annual precipitation (mm) (Bio12, 3.5%), and precipitation of the driest month (mm) (Bio14, 0.2%) were also substantial contributors to the model, with a cumulative contribution of 4.9% from these three variables (Figure 5).

**Figure 6:**
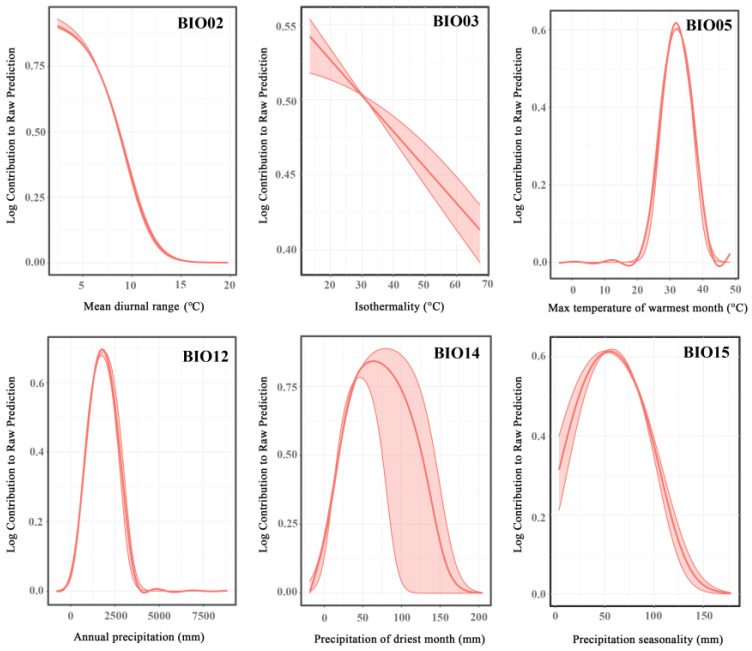
Response curves for representative variables. The vertical axis represents the probability of presence for *Ar. subalbatus,* and the horizontal axis represents the variation range of the corresponding variable.

The impact of bioclimatic variables on the probability of *Ar*. *subalbatus* occurrence is shown in Figure 6. The optimal range of Bio2 occurred between 3.867 °C and 18.306 °C. If Bio3 < 17.972 °C, the probability of presence decreased with the increase in the temperature. The impact of Bio5\Bio12\Bio14 on *Ar*. *subalbatus* increased at first and then showed a decreasing trend. The optimum range of Bio15 occurred at 18.271 mm–163.191 mm.

### 3.5. Potential Suitable Areas for Ar. subalbatus under Future Climate Scenarios

**Figure 7:**
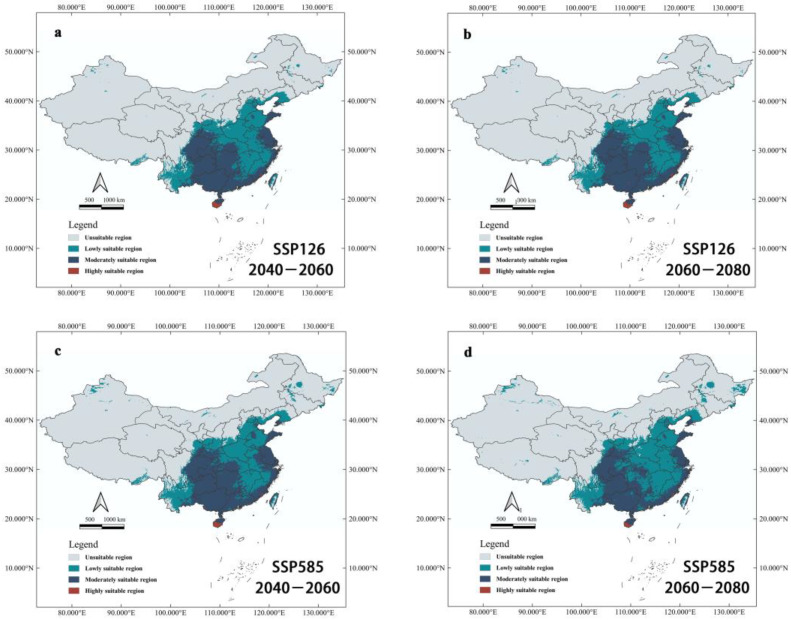
Habitat suitability of *Ar*. *subalbatus* under future climate change scenarios. (**a**) Potentially suitable areas to distribution of *Ar*. *subalbatus* within a context of climate change for 2050s (2040–2060), under the SSP126 scenario; (**b**) Potentially suitable areas to distribution of *Ar*. *subalbatus* within a context of climate change for 2070s (2060–2080), under the SSP126 scenario; (**c**) Potentially suitable areas to distribution of *Ar*. *subalbatus* within a context of climate change for 2050s (2040–2060), under the SSP585 scenario; (**d**) Potentially suitable areas to distribution of *Ar*. *subalbatus* within a context of climate change for 2070s (2060–2080), under the SSP585 scenario.

Under future climatic scenarios, the potential suitable areas of *Ar*. *subalbatus* were predicted to expand (Figure 7). Under the condition of SSP126, the potential suitable areas of the moderately and highly suitable areas will increase with the change in the time interval, and the area of the low-grade suitable area will decrease. Under the SSP585 condition, potential suitable areas changed in the other way. Low-grade suitable areas expanded dramatically, whereas suitable areas with intermediate and low levels shrank.

**Figure 8:**
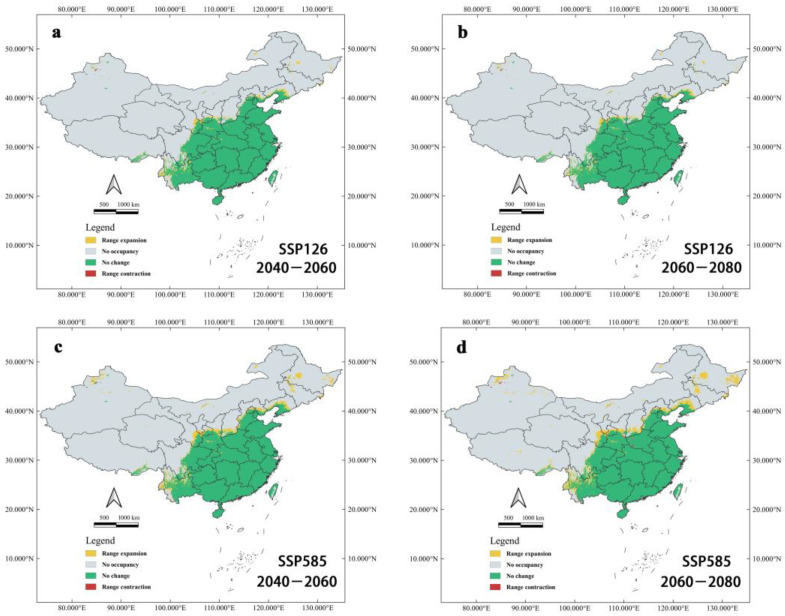
Changes to suitable habitats of *Ar*. *subalbatus* under future climate change predicted by the binary model. (**a**) Changes to suitable habitats of *Ar*. *subalbatus* within 2050s (2040–2060), under the SSP126 scenario; (**b**) Changes to suitable habitats of *Ar*. *subalbatus* within 2070s (2060–2080), under the SSP126 scenario; (**c**) Changes to suitable habitats of *Ar*. *subalbatus* within 2050s (2040–2060), under the SSP585 scenario; (**d**) Changes to suitable habitats of *Ar*. *subalbatus* within 2070s (2060–2080), under SSP585 scenario.

The binary distribution model explains the variation in suitable habitats well (Figure 8). The region of suitable habitats will significantly expand towards the northwest from the current suitable areas. Nevertheless, *Ar*. *subalbatus* will still mostly exist in the south of the Yanshan Mountains. Under SSP126 and SSP585 conductions, future suitable habitats’ expansion of *Ar*. *subalbatus* will take place in several provinces (Tibet, Xinjiang, Qinghai, Ningxia, Inner Mongolia, Yunnan, Sichuan, Gansu, Shaanxi, Shanxi, Hebei, Jilin, Heilongjiang, and Liaoning) within 2050s and 2070s. Qinghai will be the new suitable habitat in the future (2050s and 2070s). Sporadic regions of northeastern and northwestern China may develop into suitable habitats for *Ar*. *subalbatus* in all future climate scenarios.

The area of potentially suitable habitats for *Ar*. *subalbatus* in the future was also calculated according to the binary model (Table 2). Under different future climate scenarios, the current suitable area of *Ar*. *subalbatus* will remain stable. The area of suitable habitats for expansion (Figure 8) will increase over time (2050s and 2070s). According to the current study, suitable habitats’ area loss was negligible in comparison to its area increase. Under SSP585-2070s, the suitable area of *Ar*. *subalbatus* will expand to a maximum of about 372,929.9796 km^2^, which is 10.14% of the current suitable area covered.

### 3.6. Centroid Shift and Potential Suitable Areas

**Figure 9:**
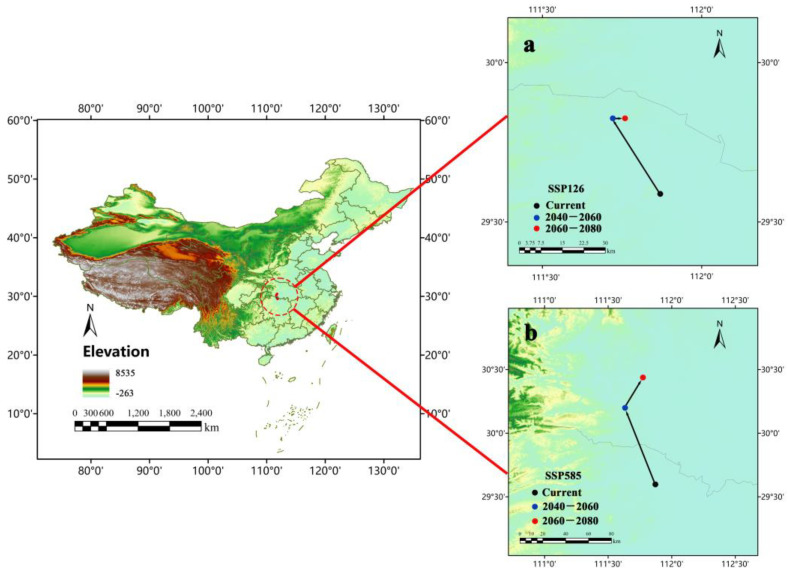
Changes of environmental suitability centroid for *Ar*. *subalbatus* within the years 2050s (2040–2060) and 2070s (2060–2080), under the (**a**) SSP126 and (**b**) SSP585 scenario.

Jinshi (111°52′11.648″ E, 29°35’32.732″ N), a city in Hunan province, was the centroid of *Ar*. *subalbatus* distribution under the current climatic conditions (Figure 9). The centroid was predicted to move toward the northeast under SSP585 (the 2050s: 111°37′50.058″ E, 30°11′57.017″ N; the 2070s: 111°46′18.177″ N). Under SSP126, the centroid may move toward the northeast (the 2050s: 111°45′18.273″ E, 29°49′32.371″ N) and then east (the 2070s: 111°43′18.788″ E, 29°49′32.371″ N). The results suggested the expansion of suitable habitats for *Ar*. *subalbatus* may be more toward the north.

**Figure 10:**
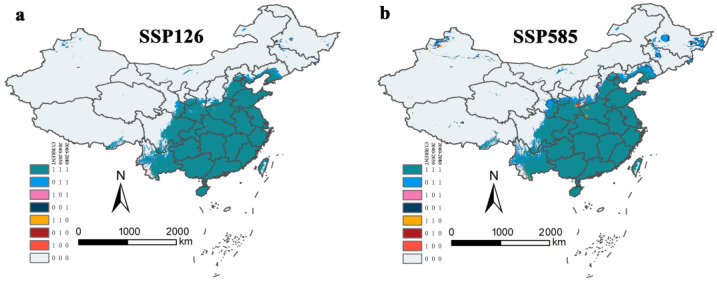
Changes to suitable areas under the (**a**) SSP126 and (**b**) SSP585 scenario. Eight colors refer to eight situations that occurred in specific locations. Green (1, 1, 1) area refers to a relatively stable region that is suitable for *Ar. subalbatus*; yellow (1, 1, 0) and orange (1, 0, 0) areas refer to a threatened region that will no longer be suitable in the 2050s or 2070s; blue (0, 1, 1) and dark-blue (0, 0, 1) areas refer to a promising region in which conditions will become suitable from an unsuitable state in the 2050s or 2070s; grey (0, 0, 0) areas remain absent throughout the entire time period. Pink (1, 0, 1) and dark-red (0, 1, 0) areas indicate areas of momentary fluctuations among the three timespans.

Most current suitable areas of *Ar*. *subalbatus* were predicted to remain suitable, but parts of Sichuan, Tibet, Gansu, Shaanxi, Shanxi, Hebei, Liaoning, and south Ningxia might face unstable changes in suitability in the 2050s and recovery in the 2070s under two SSPs (SSP 126 and SSP 585) (Figure 10). The stable area (1, 1, 1) did not change much under different SSPs, and the non-suitable area (0, 0, 0) decreased by 91,431.79 km^2^ (SSP126) and 3735.00787 km^2^ (SSP585) (Table 3). The trend of change showed the same pattern, but the degree of change was different in the two SSPs and periods. The suitable regions present in parts of Sichuan, Yunnan, Gansu, Ningxia, Shaanxi, Shanxi, Hebei, and Liaoning provinces were the primary areas in which the expected suitable areas expanded. In addition, Xinjiang, Qinghai, Inner Mongolia, Jilin, and Heilongjiang were also predicted to be suitable areas for *Ar*. *subalbatus*. The loss of suitable areas for *Ar*. *subalbatus* might occur primarily in Shaanxi, Shanxi, Yunnan, Sichuan, and Henan. The suitable reduced area (1, 0, 0) was 5496.802 km^2^ (SSP126) and 26,192.03 km^2^ (SSP585). In addition, the suitable reduced area (1, 1, 0) was 2208.117 km^2^ (SSP126) and 15,644.75 km^2^ (SSP585). Furthermore, areas indicating areas of momentary fluctuations among the three timespans (0, 1, 0) were 2208.117 km^2^ (SSP 126) and 15,644.75 km^2^ (SSP585), and areas (1, 0, 1) were 249.004 km^2^ (SSP126) and 46.98122 km^2^ (SSP585) (Table 3).

## 4. Discussion

Although *Ar. subalbatus* has been proven to be a vector of a variety of pathogens, including several pathogens such as filariasis and the Japanese encephalitis virus [27,28], it has not received enough attention, and systematic research has not been carried out [64]. Recent studies have revealed that *Ar. subalbatus* is a new potential vector for the transmission of ZIKV, posing new challenges for the global prevention of ZIKV [18,23]. In fact, the risk of potential arboviruses’ (not just Zika) transmission may expand if the neglected vectors are still underrated. In this study, we predicted *Ar. subalbatus* will rapidly spread to all the provinces of China in the future and generated habitat suitability maps based on the MaxEnt model under current and future (the 2050s and 2070s) climatic conditions. Habitat suitability maps generated here will help predict to how the distribution of *Ar. Subalbatus* will change in the future and to focus attention on areas that can be prioritized for monitoring. Our research provided basic data on *Ar. subalbatus* for assessing the arboviruses’ epidemic risk.

The geographic range and abundance of insects were greatly affected by climate change [52]. The distribution, dispersion, and adaptation of insects may all be directly influenced by climatic variables including temperature, precipitation, and humidity [52,65]. We also proved that *Ar. subalbatus* distribution is significantly influenced by temperature and precipitation. In this study, response curves were utilized to evaluate the impact of various climatic factors on the probability that *Ar. subalbatus* would emerge. The results showed that 96.1% of the variance was accounted for by temperature parameters (Bio3, Bio2, and Bio5), whereas precipitation (Bio12, Bio14, and Bio15) contributed just 3.9%. Among all climate factors, bio2 (mean diurnal range) played the most important role, contributing 85.2%, and its variation range is 3.867 °C–18.306 °C. In the range of Bio2, the suitable probability declines as the value rises. In China, the north and west have higher mean diurnal ranges (Bio2) than the south and west [66]. The highest value (above 18 °C) appears in Xinjiang, Gansu, Qinghai, Tibet, etc. [66]. In fact, *Ar. subalbatus* is currently distributed in all provinces in China except Xinjiang, western Gansu, Qinghai, most of Tibet, Inner Mongolia, Liaoning, Heilongjiang, and Jilin. The distribution of *Ar. subalbatus* basically shows characteristics similar to the mean diurnal ranges in China. The implications of the mean diurnal range and vector distribution require further validation, but we may provide a good start. Relatively little research has been performed on *Ar. Subalbatus*. In a previous study [39], it was also found that two factors (temperature and relative humidity variation) may be important for the phenology of adult *Ar. subalbatus*, as the species was only found at temperatures above 14 °C and relative humidity above 65%. In addition, precipitation provides larval habitat and positively affects the activity and number of *Ar. Subalbatus* by impacting its egg hatching and diapause [39,67,68,69]. Our data showed that the contribution of precipitation is 3.9%, which may be related to precipitation that can affect the number of *Ar. subalbatus*, but its distribution range is mainly affected by temperature (especially the influence of Bio2).

Under the climate change scenarios of SSP126 and SSP585 compared to present climatic conditions, we can observe the expansion trend of suitable habitats for *Ar. subalbatus* in the 2050s and 2070s. The predictions under the SSP585-2070s scenario showed the worst results, followed by SSP585-2050s. Under SSP 585-2070s, the suitable area for *Ar. subalbatus* will reach a maximum value of about 4,179,713 km^2^, which is 1.137 times the current area. There are two types of new suitable habitats in different climatic conditions. The first type is continuous distribution expansion, and the expansion trend of suitable habitats for *Ar. subalbatus* is northward in Yunnan, Sichuan, Gansu, Ningxia, Shaanxi, Shanxi, Hebei, and Liaoning. Under the SSP585 and SSP126, the expansion can extend to southern Liaoning in mainland China. The second type (sporadic distribution expansion) showed that the new potential suitable areas are scattered in Xinjiang, Inner Mongolia, Qinghai, Heilongjiang, and Jilin for the first timespan, which do not border the current suitable habitat. The first type of expansion demands strengthening the monitoring of the occurrence and breeding of vectors. The second type deserves more complex monitoring and needs to pay attention to tourism and trade in addition to the requirements of the first type, because the frequency of *Ar. subalbatus* occurrence in new potential suitable areas will probably increase with increasing global trade and travel [66]. Furthermore, under all future modeling scenarios of this study, the distribution range of *Ar. subalbatus* may expand and provide more new suitable habitats, which might potentially put a bigger human population at risk.

As with studies that predict the impact of climate change on other mosquito species, Maxent models have some limitations and uncertainties in predicting future species distributions [4,22,54,70,71]. The assumption used to predict suitable habitats is that the species will not experience any dispersion limitations [22]. Additionally, the influence of biological interactions is ignored. Therefore, our predictions were made under ideal conditions. The models developed in our study emphasize that climate is a key driver of mosquito distribution, and our results established that temperature plays a major role in the distribution of *Ar. subalbatus*. As these predictions were not based on field investigation, our results can only be used as a reference for future mosquito monitoring. Decision-makers must fully understand the uncertainty of model predictions and combine field investigations to create the right strategy.

## Figures and Tables

**Table 1:** Bioclimatic variables used in the model.

Bioclimatic Variable	Description	Included
Bio01	Annual mean temperature	
Bio02	Mean diurnal temperature range	Yes
Bio03	Isothermality	Yes
Bio04	Temperature seasonality	
Bio05	Max temperature of the warmest month	Yes
Bio06	Min temperature of the coldest month	
Bio07	Annual temperature range	
Bio08	Mean temperature of the wettest quarter	
Bio09	Mean temperature of the driest quarter	
Bio10	Mean temperature of the warmest quarter	
Bio11	Mean temperature of the coldest quarter	
Bio12	Annual precipitation	Yes
Bio13	Precipitation of the wettest month	
Bio14	Precipitation of the driest month	Yes
Bio15	Precipitation seasonality	Yes
Bio16	Precipitation of the wettest quarter	
Bio17	Precipitation of the driest quarter	
Bio18	Precipitation of the warmest quarter	
Bio19	Precipitation of the coldest quarter	

**Table 2:** Estimated gain, stability, and loss of suitable habitat area for *Ar. subalbatus* under future climate change scenarios.

Time Period	SSPS Scenarios	Area Change (%)			
		Range Expansion	No Change	Range Contraction	Net Change
2040–2060	SSP126	4.193%	78.385%	0.160%	4.033%
2040–2060	SSP585	7.755%	78.466%	0.079%	7.675%
2060–2080	SSP126	4.151%	78.388%	0.157%	3.994%
2060–2080	SSP585	10.144%	78.140%	0.404%	9.739%

**Table 3:** Changes to suitable areas under climate change: 0 refers to an absence of the species, and 1 refers to its presence.

Present or Absent in Different Periods			Areas under Climate Scenarios (km^2^)	
CURRENT	2045–2060	2060–2070	SSP126	SSP585
1	1	1	36,378.97	36,286.65
0	1	1	18,778.39	36,541.99
1	0	1	249.004	46.98122
0	0	1	17,030.69	15,327.62
1	1	0	2208.117	15,644.75
0	1	0	17,970.32	26,192.03
1	0	0	5496.802	26,192.03
0	0	0	91,431.79	3735.007

## Data Availability

The data presented in this study are available in this article. For the data provided in this study, see Section 2.1 and Section 2.2.
